# Use of a New High Resolution Melting Method for Genotyping Pathogenic *Leptospira* spp.

**DOI:** 10.1371/journal.pone.0127430

**Published:** 2015-07-08

**Authors:** Florence Naze, Amélie Desvars, Mathieu Picardeau, Pascale Bourhy, Alain Michault

**Affiliations:** 1 Department of Microbiology, CHU de La Reunion, Saint-Pierre, La Réunion, France; 2 Unité de Biologie des Spirochètes, National Reference Center and WHO Collaborating Center for Leptospirosis, Institut Pasteur, Paris, France; Cornell University, UNITED STATES

## Abstract

**Background:**

Leptospirosis is a worldwide zoonosis that is endemic in tropical areas, such as Reunion Island. The species *Leptospira interrogans* is the primary agent in human infections, but other pathogenic species, such as *L*. *kirschner* and *L*. *borgpetersenii*, are also associated with human leptospirosis.

**Methods and Findings:**

In this study, a melting curve analysis of the products that were amplified with the primer pairs lfb1 F/R and G1/G2 facilitated an accurate species classification of Leptospira reference strains. Next, we combined an unsupervised high resolution melting (HRM) method with a new statistical approach using primers to amplify a two variable-number tandem-repeat (VNTR) for typing at the subspecies level. The HRM analysis, which was performed with ScreenClust Software, enabled the identification of genotypes at the serovar level with high resolution power (Hunter-Gaston index 0.984). This method was also applied to *Leptospira* DNA from blood samples that were obtained from Reunion Island after 1998. We were able to identify a unique genotype that is identical to that of the *L*. *interrogans* serovars Copenhageni and Icterohaemorrhagiae, suggesting that this genotype is the major cause of leptospirosis on Reunion Island.

**Conclusions:**

Our simple, rapid, and robust genotyping method enables the identification of *Leptospira* strains at the species and subspecies levels and supports the direct genotyping of *Leptospira* in biological samples without requiring cultures.

## Introduction

Pathogenic *Leptospira* bacteria are agents of leptospirosis, one of the most widespread zoonoses worldwide[1]. *Leptospira* spp. can infect a wide range of animal species, which serve as maintenance hosts, but rodents are the most important reservoir of *Leptospira* spp. Humans typically become infected via contact with urine-contaminated soil or water or, less frequently, by direct contact with the tissues or urine of animals[2]. *Leptospira* spp. have been subdivided and classified into more than 260 serovars that are grouped into serogroups based on shared antigens [1]. Molecular studies have helped to determine a classification system based on genetic similarities which is currently used in conjunction with antigenic classification [3]. This molecular taxonomy system classifies *Leptospira* into 21 genomospecies [4,5]: nine pathogenic species, five intermediate species, and seven saprophytic species [3]. In regions of endemicity, the identification of the infecting strain can help to determine the infectious animal source, and thus aid in the implementation of control measures [2]. Various molecular techniques have been described for studying the molecular epidemiology of *Leptospira*, including pulsed-field gel electrophoresis (PFGE) [6], restriction enzyme analysis (REA) [7], ribotyping [8], randomly amplified polymorphic DNA (RAPD) [9], restriction fragment length polymorphism (RFLP) [10], fluorescent amplified fragment length polymorphism (FAFLP) [11], 16S rRNA sequencing [12] and several PCR-based approaches. Unfortunately, all of these methods suffer from significant drawbacks [13]. In recent years, two genotyping methods, multiple loci variable number of tandem repeats analysis (MLVA) [13] and multilocus sequence typing (MLST) [14], have emerged as noteworthy PCR-based genotyping techniques, but one major limitation of these methods is their reliance on bacterial cultures to generate a significant amount of high-pure DNA [14,15]. Moreover, MLVA requires the determination of the PCR product size, which requires agarose gel electrophoresis, a time-consuming technique with a poor resolution of amplicon size. MLST requires high-throughput sequencing and bioinformatics, but provides a portable, reproducible, and scalable typing system[14,16].

High-resolution melting (HRM) analysis [17], which measures the melting temperatures of amplicons in real time using a fluorescent DNA-binding dye, is a closed-tube, non-sequencing-based system for genotyping and mutation scanning[17,18]. This method has emerged as a valuable tool for the rapid testing of diverse biological specimens and tissues for the presence of micro-organisms and for the differentiation of *Brucella* spp. genetic variants [19], for example, *Chlamydiaceae* [20], *Mycoplasma pneumoniae* [21], *Leishmania* [22], *Bordetella pertussis* [23], *Staphylococcus aureus* [24], *Bacillus anthracis* [25], *Mycoplasma synoviae*[26], *Pseudomonas aeruginosa [27]* adenovirus serotypes [28], and *Aspergillus* species [29]. HRM has even been used for the identification of members of the *Anopheles funestus* group [30]. Tulsiani *et al*.[31] developed a random amplification of polymorphic DNA (RAPD) method associated with HRM analysis (RAPD-HRM) using 13 previously published RAPD primers to genotype 10 *Leptospira* strains. Traditional HRM curves are difficult to interpret, thus, the interpretation of HRM results can be arbitrary. In this study, we evaluated the potential of an alternative method based on unsupervised high-resolution melting curve (HRM) analysis using ScreenClust HRM (Qiagen, Courtabeuf, France) and examined the ability of this new method to type *Leptospira* from human Reunion Island specimens rapidly and easily.

## Materials and Methods

### *Leptospira* reference strains and human samples

Forty-nine *Leptospira* reference strains (Table 1) and seven in vitro cultured strains obtained from human patients from Mayotte [32] (*L*. *interrogans* sg. Pyrogenes, *L*. *borgpetersenii* sg. Mini, *L*. *borgpetersenii* sg. Pomona, *L*. *kirchneri* sg. Mini/Hebdomadis, *L*. *kirchneri* sg. Grippotyphosa, *L*. *mayottensis* sg. Pyrogenes/Ballum [33]) were provided by the National Reference Center for Leptospirosis, Institut Pasteur (Paris, France).

**Table 1:** Determination of species and genotypes by real-time PCR with different sets of primers.

Species	Serogroup	Serovar	Strain	Tm LFB1F/R	Tm G1/G2	Cluster VNTR-4bis	ClusterVNTR-Lb4	Cluster VNTR Lb5	Profile	Genotype
*L*. *interrogans* *	Hebdomadis	Hebdomadis	Hebdomadis	80.68	78.62	2	na	5	2–5	1
*L*. *interrogans* *	Icterohaemorrhagiae	Copenhageni	Wijinberg	80.35	78.70	5	na	1	5–1	2
*L*. *interrogans* *	Australis	Australis	Ballico	80.87	78.05	1	na	7	1–7	3
*L*. *interrogans* *	Autumnalis	Autumnalis	Akiyami A	80.57	77.95	10	na	3	10–3	4
*L*. *interrogans* *	Canicola	Canicola	Hond Utrech IV	80.73	78.5	1	na	4	1–4	5
*L*. *interrogans* *	Pyrogenes	Pyrogenes	Salinem	80.82	78.72	4	1	2	4–2	6
*L*. *interrogans* *	Pomona	Pomona	Pomona	80.90	78.45	7	na	3	7–3	7
*L*. *interrogans* *	Icterohaemorrhagiae	Icterohaemorrhagiae	Verdun	80.48	78.88	5	na	1	5–1	2
*L*. *interrogans*	Grippotyphosa	Grippotyphosa	Andaman	80.75	78.32	15	na	3	15–3	8
*L*. *interrogans*	Canicola	Kuwait	136/2/2	80.77	78.50	16	na	4	16–4	9
*L*. *interrogans*	Canicola	Schueffneri	Vleermuis 90 C	80.83	78.53	2	na	2	2–2	10
*L*. *interrogans*	Pyrogenes	Biggis	Biggs	81.1	78.48	11	na	2	11–2	11
*L*. *interrogans*	Sejroe	Haemolytica	Marsh	80.82	78.85	3	na	2	3–2	12
*L*. *interrogans*	Pyrogenes	Guaratuba	An 7705	81.00	78.78	7	na	3	7–3	7
*L*. *interrogans*	Canicola	Broomi	Patane	80.82	78.73	4	na	7	4–7	13
*L*. *interrogans*	Australis	Fugis	Fudge	80.85	78.22	7	na	6	7–6	14
*L*. *interrogans*	Canicola	Sumneri	Sumner	80.77	78.88	12	na	1	12–1	15
*L*. *interrogans*	Pomona	Kennewicki	LT 10–26	80.98	78.85	13	na	1	14–1	16
*L*. *interrogans*	Icterohaemorrhagiae	Birkini	Birkin	80.87	78.15	8	na	1	8–1	17
*L*. *interrogans*	Canicola	Jonsis	Jones	80.90	78.77	2	na	1	2–1	18
*L*. *interrogans*	Sejroe	Ricardi	Richardson	80.68	78.48	14	na	2	14–2	19
*L*. *interrogans*	Djasiman	Djasiman	Djasiman	80.42	78.15	9	na	2	9–2	20
*L*. *interrogans*	Djasiman	Gurungi	Gurung	80.48	78.58	17	na	3	17–3	21
*L*. *interrogans*	Sejroe	Hardjo	Hardjoprajitno	80.80	78.80	1	na	7	1–7	3
*L*. *interrogans*	Icterohaemorrhagiae	Lai	Lai	80.85	78.82	6	2	2	6–2	22
*L*. *interrogans*	Australis	Bratislava	Jez-Bratislava	80.78	78.33	1	na	7	1–7	3
*L*. *interrogans*	Icterohaemorrhagiae	Copenhageni	Fiocruz L1-130	80.45	78.77	5	na	1	5–1	2
*L*. *interrogans* (Average Tm ± S.D.)				80.75 (± 0.19)	78.55 (± 0.27)					
*L*. *borgpetersenii* *	Ballum	castellonis	Castellonis 3	83.00	80.65	na	1	2	1–2	1
*L*. *borgpetersenii* *	Sejroe	sejroe	M84	83.01	80.75	na	1	1	1–1	2
*L*. *borgpetersenii* *	Tarassovi	tarassovi	perepelicin	82.95	80.90	na	2	3	2–3	3
*L*. *borgpetersenii* *	Sejroe	hardjobovis	sponselee	82.73	80.87	na	3	3	3–3	4
*L*. *borgpetersenii*	Hebdomadis	Jules	Jules	83.35	81.25	na	1	2	1–2	1
*L*. *borgpetersenii*	Javanica	Ceylonica	Piyasena	83.25	81.00	na	2	1	2–1	5
*L*. *borgpetersenii*	Mini	Mini	Sari	83.30	81.03	na	3	4	3–4	6
*L*. *borgpetersenii*	Hebdomadis	Nona	Nona	83.40	81.20	na	2	1	2–1	5
*L*. *borgpetersenii*	Hebdomadis	Worsfoldi	Worsfold	82.50	81.25	na	4	5	4–5	7
*L*. *borgpetersenii*	Pyrogenes	Hamptoni	Hampton	82.63	80.75	na	5	6	5–6	8
*L*. *borgpetersenii* (Average Tm ± S.D.)				83.00 (± 0.31)	81.10 (± 0.31)					
*L*. *kirschneri* *	Grippotyphosa	Grippotyphosa	Moskva V	81.42	na	5	na	3	5–3	1
*L*. *kirschneri* *	cynopteri	Cynopteri	3522C	81.63	na	4	na	3	4–3	2
*L*. *kirschneri* *	Mini	Hebdomadis	200801925	81.65	na	5	na	2	5–2	3
*L*. *kirschneri*	Icterohaemorrhagiae	Bogvere	LT 60–69	81.90	na	1	na	1	1–1	4
*L*. *kirschneri*	Icterohaemorrhagiae	Ndambari	Ndambari	82.08	na	1	na	2	1–2	5
*L*. *kirschneri*	Bataviae	Djatzi	HS 26	81.90	na	1	na	2	1–2	4
*L*. *kirschneri*	Icterohaemorrhagiae	Mwogolo	Mwogolo	81.90	na	1	na	1	1–1	6
*L*. *kirschneri*	Grippotyphosa	Ratnapura	Wumalasena	82.40	na	3	na	2	3–2	4
*L*. *kirschneri*	Icterohaemorrhagiae	Ndahambukuje	Ndahambukuje	81.92	na	1	na	1	1–1	6
*L*. *kirschneri*	Canicola	Galtoni	LT 1014	82.35	na	3	na	2	3–2	7
*L*. *kirschneri*	Pomona	Tsaratsovo	B 81/7	81.90	na	2	na	1	2–1	
*L*. *kirschneri* (Average Tm ± S.D.)				81.90 (± 0.28)	na					
*L*. *noguchii* *	Panama	Panama	CZ214K	81.48	79.20					

*Sixteen reference strains were used to develop the typing method.

Forty-two *Leptospira* DNA samples were extracted from the sera of leptospirosis-positive patients who were diagnosed between January 2008 and March 2012 at the Groupe Hospitalier Sud Réunion (Saint-Pierre, Reunion Island, France). The PCR-based microbiological diagnoses used the primers G1/G2 [34] before 2010 and lfb1 F/R [35] after 2010. The samples were stored at -80°C.

The DNA extractions from the in vitro cultured strains and sera were performed using the NucliSens easyMAG system (BioMérieux, Marcy l’Etoile, France). The concentration of the *Leptospira* DNA was normalized using the quantification analysis module of a RotorGene 6000 cycler (Qiagen, Courtabeuf, France). To normalize the input template concentration for the strains, the Ct (threshold cycle) was set to be between 27 and 30 cycles.

### PCR amplification and analysis

We tested seven primer pairs specific to *Leptospira* on 16 *Leptospira* reference strains: secY IV F/R [36], lgB F/R [37], A/B [38], lfb1 F/R [35], G1/G2 [34], lpL32 F/R [39], and lpL41 F/R [14], with the aim of selecting specific primers for *Leptospira* species identification. In all the PCRs, *L*. *biflexa* sv. Patoc was used as a negative control.

The sensitivity of each PCR using the selected primer(s) was evaluated by performing PCRs on 10-fold serial dilutions of the DNA extracted from three in vitro cultured strains from Mayotte: *L*. *interrogans* sg. Pyrogenes, *L*. *borgpetersenii* sg. Mini, and *L*. *kirchneri* sg. Mini/Hebdomadis. The quantification of the leptospires was based on genomic DNA mass, taking into consideration that the size of the genome of the *L*. *interrogans* strain Fiocruz L1-130 is 4.6 Mb (1 genome is approximately 5 fg). The end-point detection limit was determined by 10 repetitions of the measurement of the last positive point with 100% amplification.

We also cloned a plasmid DNA carrying the G1/G2 gene of *L*. *interrogans* sv. Copenhageni strain Wijinberg into the pGEM-T Easy Vector (Promega, USA).

A subspecies determination was performed on the matching reference strains (with the exception of *L*. *noguchii*), and eleven primer pairs amplifying polymorphic tandem repeat sequences were tested (VNTR 4, VNTR 7, VNTR 10, VNTR 19, VNTR 23, VNTR 31, VNTR 4bis, VNTR 7bis, VNTR 10bis, VNTR Lb4, and VNTR Lb5) [13,40,]. These VNTR primers were used in a previously published MLVA study and exhibited a noteworthy discriminatory power for the identification of the serovars of *L*. *interrogans*, *L*. *borgpetersenii*, and *L*. *kirschneri* [40].

The reproducibility of our method (which refers to the variation in our results between runs) was evaluated by ten repetitions of PCRs with the selected primer(s) for three strains (*L*. *kirschneri* sv. Grippotyphosa str. Moskva V, *L*. *borgpetersenii* sv. Hardjobovis str. Sponselee, and *L*. *interrogans* sv. Icterohaemorrhagiae str. Verdun). For each strain, if the species identification was identical in the ten repetitions, then the reproducibility was determined to be 100%.

The PCRs were performed using a Type-IT HRM PCR Qiagen Kit (Qiagen, Courtabeuf, France) on a RotorGene 6000 system (Qiagen, Courtaboeuf, France). The 20 μl reactions contained 10 μl of mix 2X master mix HRM, with a 0.7 μM final concentration of each primer (TibMolBiol, Berlin, Germany), and 5 μl of the extracted nucleic acid solution. The following amplification protocol was used: denaturation at 95°C for 5 min, 45 cycles at 95°C for 10 s, 55°C for 30 s, and 72°C for 10 s. These conditions were used for all the primer pairs. For the species determination, a melting curve analysis determined the melting temperature (Tm). To test the reproducibility of the Tm determination, three strains of different species were tested in 10 separate runs with the selected primers. For the subspecies determination, after PCR cycling with the VNTR primers, the samples were heated from 65°C to 95°C with continuous acquisition. For each VNTR locus, a normalization region of the melting curve was selected to improve the analysis per the recommendations of the Rotor-Gene ScreenClust HRM Software User Guide (Qiagen, Courtabeuf, France). Two normalization regions were selected: one before and one after the melting curve transition. The highest fluorescence value was 100 and the lowest fluorescence value was zero. The data were analyzed using ScreenClust HRM (Qiagen, Courtabeuf, France) in unsupervised mode (i.e., there were no known controls for each cluster and the number of clusters was unknown). The unsupervised mode was also employed to analyze *Leptospira* DNA fluorescence data obtained with human samples, and the results from the control strains were employed as pseudo-unknowns. The data were analyzed by ScreenClust using the principal component analysis statistical method [41], which enabled the maximum separation of genotypes.

A cluster plot was built in three dimensions (principal components) and ellipses representing each cluster were drawn. The probability of each sample fitting into a specific cluster was calculated and required to be at least 0.7. The typicality, which measures how well a sample falls within the cluster to which it has been assigned, was also given for each sample and was required to be at least 0.05.

The genotype [42] of each *Leptospira* DNA was determined by combining the results of the two clusters that were obtained with the two VNTR primers selected by the species in the subspecies characterization.

To evaluate the ability of the tested primers to correctly identify the *Leptospira* species, a Mann-Whitney test was used to determine whether the difference in the mean Tm was significant between species.

The typeability of each set of primers for each species of *Leptospira* (i.e., the proportion of strains that were assigned a type by the typing system) was determined by using the following formula: T = Nt/N, where Nt is the number of strains that were assigned a type and N is the number of strains that were tested [5].

To evaluate the discriminatory power of the VNTR primers, the Simpson diversity index (DI) was used as described by Hunter and Gaston[43].

1). The polymorphism is considered to be high when the Simpson diversity index is greater than 95%[42,44].

## Results

### Determination of species

**Fig 1:**
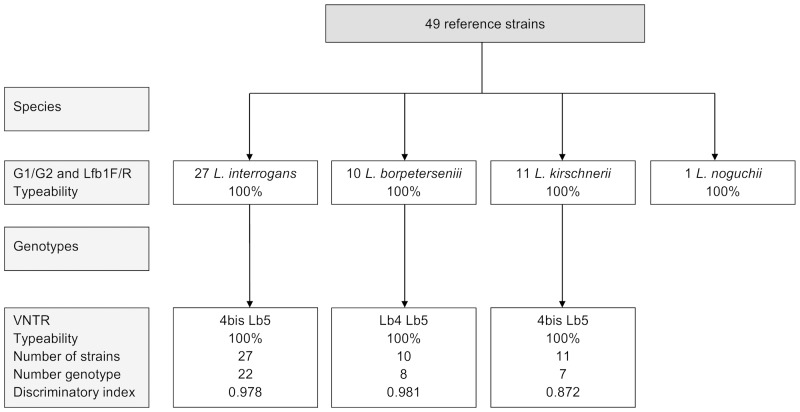
Determination of the *Leptospira* species and genotypes with reference strains.

**Fig 2:**
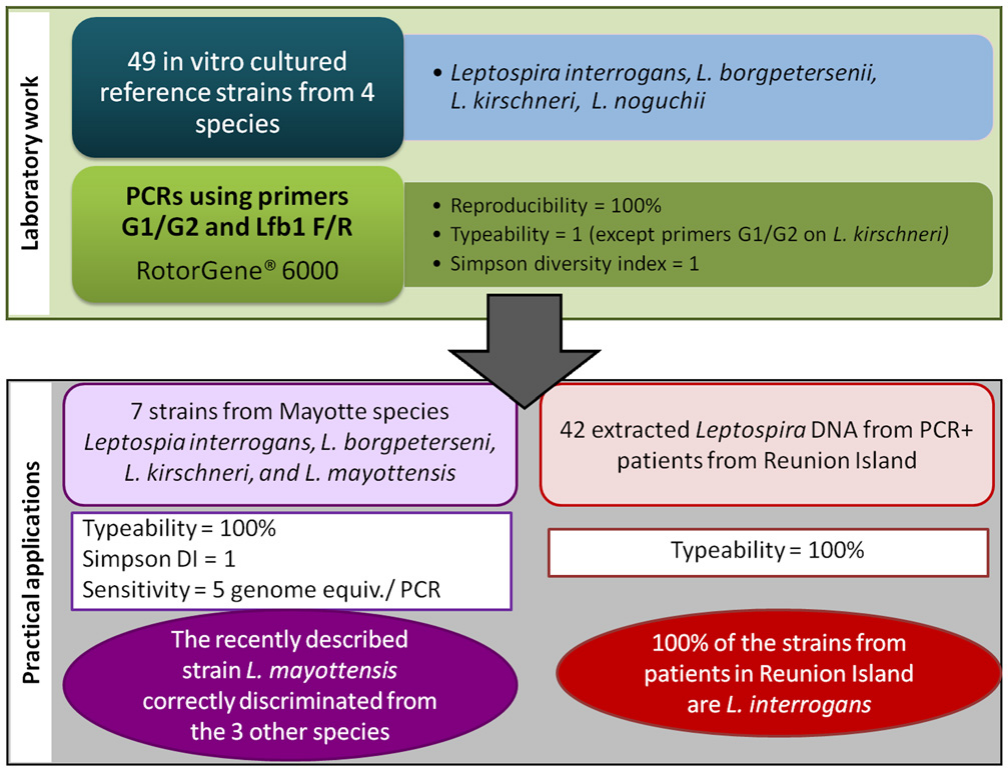
The materials and methods implemented in this study for the genotyping of *Leptospira* at the species level, and practical applications.

**Fig 3:**
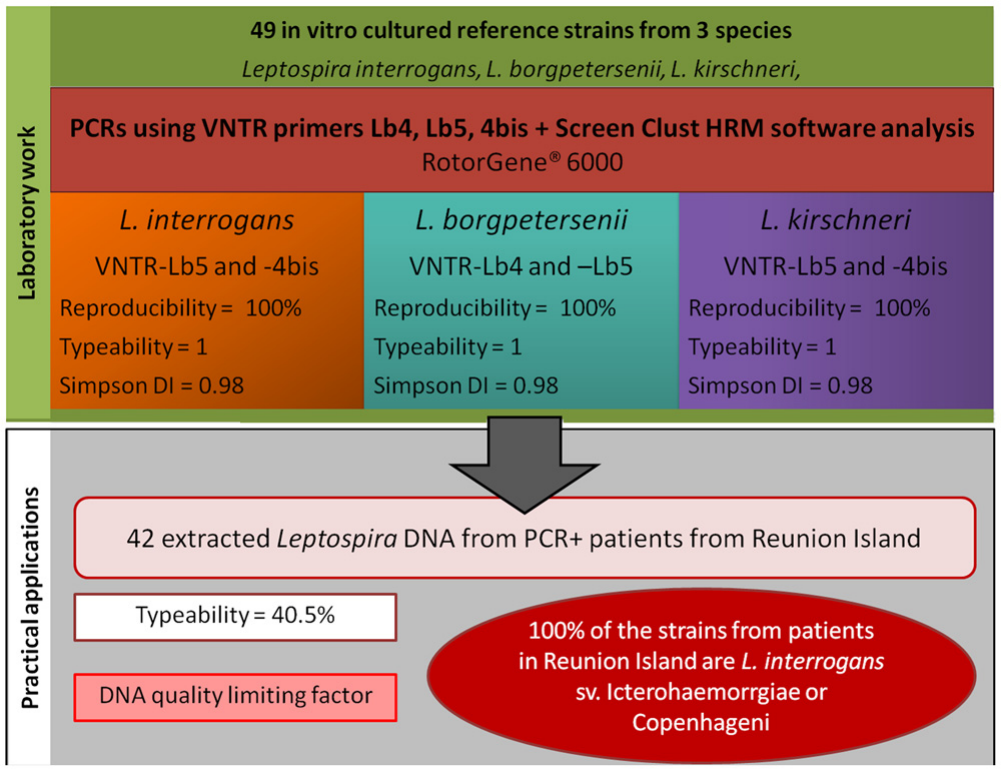
Melting curve analysis of pathogenic *Leptospira* strains after real-time PCR amplification using the G1/G2 and LFb1F/R primer sets.

Our results are shown in Table 1 and Figs 1, 2 and 3. In all the runs, the negative control, a DNA extract from an in vitro culture of *L*. *biflexa* sv. Patoc, did not show amplification with lfb1F/R nor G1/G2. Seven primers were tested on 16 strains to characterize each species, including *L*. *interrogans*, *L*. *borgpetersenii*, *L kirschneri*, and *L*. *noguchii*. Only the association of the primer pairs lfb1F/R and G1/G2 resulted in a species-specific Tm (except *L kirschneri*, which had no amplification with G1/G2) and allowed for the discrimination of the four species (p<0.001); thus, these two primer pairs were selected for further analysis.

The reproducibility (evaluated on ten runs) was 100% for the three tested strains using the two primer pairs lfb1F/R and G1/G2.

The coefficient of variation of the Tm was 0.19% for strain *L*. *kirschneri* sv. Grippotyphosa str. Moskva V, 0.4% for *L*. *borgpetersenii* sv. Hardjobovis str. Sponselee and 0.22% for *L*. *interrogans* sv. Icterohaemorrhagiae str. Verdun with lfb1 F/R and 0.5% for *L*. *borgpetersenii* sv. Hardjobovis str. Sponselee and 0.45% for *L*. *Interrogans* sv. Icterohaemorrhagiae str. Verdun with G1/G2.

Among the seven strains from Mayotte, those from the species *L*. *interrogans*, *L*. *borgpetersenii*, and *L*. *kirschneri* were well discriminated by the primer pairs lfb1F/R and G1/G2. *L*. *mayottensis* was not amplified by G1/G2, similar to *L*. *kirschneri*, but the Tm resulting from the lfb1 F/R amplification was significantly different for *L*. *mayottensis* (Tm 83.76±0.3) and *L*. *kirschneri* (81.90 ± 0.28).

The sensitivity was evaluated for the two selected pairs of primers, lfb1F/R and G1/G2, using three strains. For each PCR assay (with the exception of G1/G2, which did not amplify the *L*. *kirschneri* strain) the two sets of primers showed that 100% positive amplification was obtained for an average of 5 genome equivalents per PCR (Ct ≈ 37 cycles).

Because one leptospire contains an average of five genome equivalents [49], our analytical sensitivity was calculated to be 1 bacterium/μl. This result was confirmed by using the plasmid DNA of the G1/G2 gene of *L*. *interrogans* sv. Copenhageni strain Wijinberg because we had obtained a limit of detection of 25 copies per PCR for *L*. *interrogans* sg. Pyrogenes.

The limits of detection on plasma were assessed using DNA that was extracted, diluted from identical strains and spiked. The limit of detection on the spiked plasma was estimated to be 5 genome equivalents per PCR, which was similar to that of the two sets of primers on the different strains and the extracted unspiked DNA.

### Genotype determination of *Leptospira* reference strains using ScreenClust HRM Software

**Fig 4:**
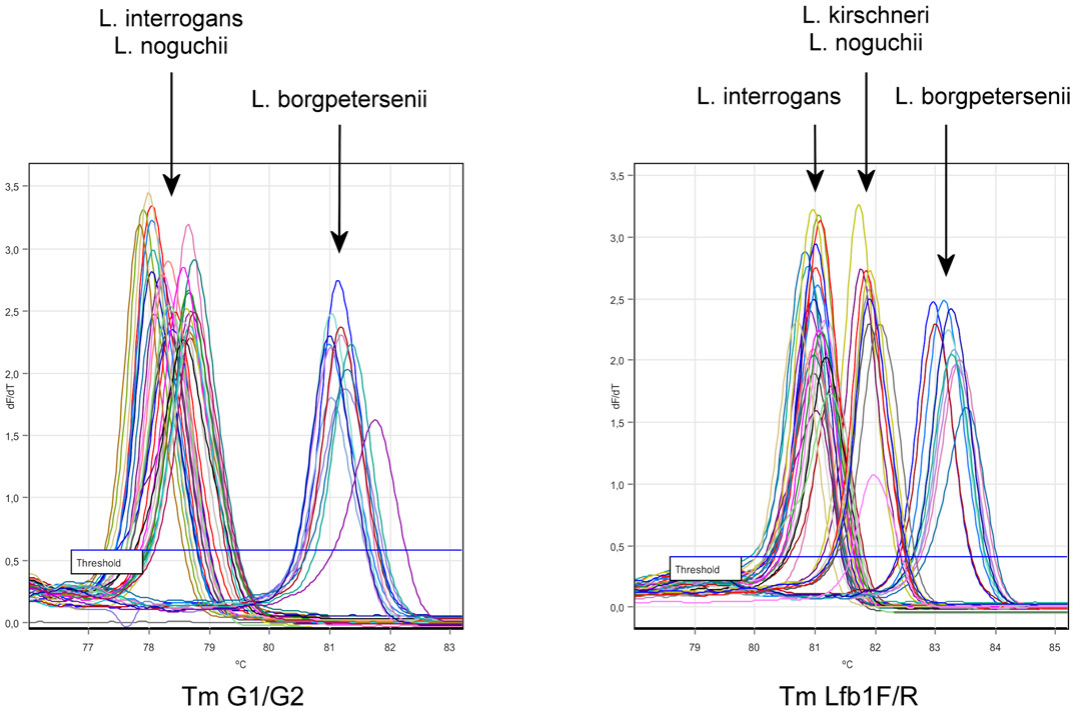
The materials and methods implemented in this study for the genotyping of *Leptospira* at the subspecies level, and practical applications.

**Fig 5:**
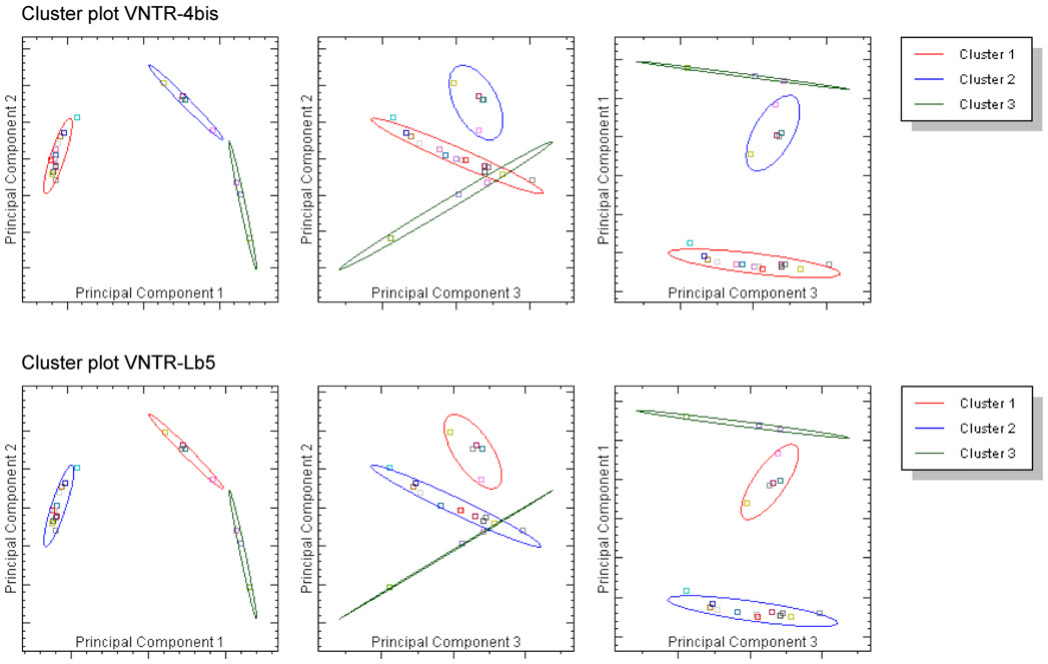
Cluster plot obtained after the HRM analysis using the VNTR-4bis and VNTR-Lb5.

Of the 11 primer sets tested, three VNTR primer sets were considered to be noteworthy for discriminating the strains and were considered further: VNTR-Lb4, VNTR-4 bis and VNTR-Lb5 (Table 1, Figs 1, 4 and 5). Two VNTR primer pairs per species were used to genotype *Leptospira* at the subspecies level. Using these three VNTR primers, the probability of a strain belonging to a specific cluster was ≥ 0.95, and the typicality was ≥ 0.05 for all the strains.

#### L. interrogans

Using VNTR-Lb4, the typeability value obtained for the 27 *L*. *interrogans* strains tested was low (0.074); consequently, VNTR-Lb4 was not employed further. The typeability values for VNTR-4bis and -Lb5 were both 1; thus, these two VNTRs were selected for further study, and their normalization temperatures were 83.7–89.3°C and 84.5–88.4°C, respectively. The reproducibility of the PCR runs with the two primers VNTR-4bis and -Lb5 was 100%, which was tested on *L*. *interrogans* sv. Icterohaemorrhagiae str. Verdun.

The HRM analysis of the amplicons obtained with primers VNTR-4bis and -Lb5 was highly discriminating, with 17 and seven clusters generated, respectively, for the 27 different *L*. *interrogans* strains tested. By combining the results using VNTR-4bis and -Lb5, we found 22 genotypes in the 27 strains tested, with 19 strains having a unique genotype (DI = 0.98) (Table 1). However, some strains could not be discriminated: cluster 2 included three strains belonging to the serogroup Icterohaemorrhagiae (sv. Copenhageni str. Wijinberg, sv. Icterohaemorrhagiae str. Verdun, and sv. Copenhageni str. Fiocruz L1-130) and cluster 3 included two serovars from the serogroup Australis (sv. Australis str. Ballico and sv. Bratislava str. Jez-Bratislava) and sv. Sejroe. Moreover, cluster 7 included sv. Pomona str. Pomona and sv. Guaratuba str. An 7705.

#### L. borgpetersenii

UsingVNTR-4bis, the typeability value obtained for the 10 *L*. *borgpetersenii* strains tested was low (0.1); consequently, VNTR-4bis was not employed further. The typeability for the 10 *L*. *borgpetersenii* strains with VNTR-Lb4 and -Lb5 was 1; thus these two VNTRs were selected for further study, and their normalization temperatures were 79.8–82.9°C and 84.3–86°C, respectively.

The PCR reproducibility with the two primers VNTR-Lb4 and -Lb5 was 100%, which was tested on *L*. *borgpetersenii* sv. Hardjobovis str. Sponselee.

The HRM analysis of the amplicons obtained with primerVNTR-Lb4 generated five clusters for the ten *L*. *borgpetersenii* strains, while VNTR-Lb5 discriminated six different clusters. Thus, in the analysis of the *L*. *borgpetersenii* strains, the combination of Lb4 and -Lb5 generated eight out of 10 genotypes, with six strains having a unique genotype (DI = 0.98) (Table 1). The method could not discriminate sv. Ceylonica str. Piyasena from sg. Javanica, and sv. Nona str. Nona from sg. Hebdomadis, which were grouped into cluster 5, nor sv. Castellonis str. Castellonis 3 sg. Ballum from sv. Jules str. Jules sg. Hebdomadis, which were grouped into cluster 1.

#### L. kirschneri

Using VNTR-Lb4, the typeability value obtained for the 11 *L*. *kirschneri* strains tested was 0; consequently, VNTR-Lb4 was not employed further. The typeability using VNTR-4bis and -Lb5 was 1; thus, these two VNTRs were selected for further study, and their normalization temperatures were 81.3–83.3°C and 83.7–86.7°C, respectively.

The PCR reproducibility using the primers VNTR-4bis and -Lb5 was 100%, which was tested on *L*. *kirschneri* sv. Grippotyphosa str. Moskva V.

The HRM analysis of the amplicons obtained with the primer VNTR-4bis enabled the discrimination of five clusters for the 11 *L*. *kirschneri* strains, whereas VNTR-Lb5 discriminated three clusters within this species. A combination of these two VNTRs generated seven genotypes in the 11 *L*. *kirschneri* strains, and five strains had a unique genotype (DI = 0.87) (Table 1). Cluster 4 included three serovars of the serogroup Icterohaemorrhagiae (sv. Bogvere str. LT 60–69, sv. Mwogolo str. Mwogolo and sv. Ndahambukuje str. Ndahambukuje). Moreover, sv. Ndambari str. Ndambari from sg. Icterohaemorrhagiae and sv. Djatzi str. HS 26 from sg. Bataviae were not discriminated using our method (cluster 5), and similarly, sv. Ratnapura str. Wumalasena from sg. Grippotyphosa and sv. Galtoni str. LT 1014 from sg. Canicola, were included together in cluster 6.

Our method generated 37 genotypes in 48 reference strains. Thirty strains had a unique genotype while seven clusters contained two to three strains. Thus, the global DI of our method was 0.98.

### Application of ScreenClust software to biological samples (Table 2, Figs 2 and 3)

**Table 2:** Clusters of species and DNA extracted from sera with the VNTR 4bis and Lb5 primers shown in Fig 5.

Species	Serogroup	Serovar	Strains or DNA extracted from sera	Cluster VNTR-4bis	ClusterVNTR-Lb5	Genotype
*L*. *interrogans*	*Icterohaemorrhagiae*	*Icterohaemorrhagiae*	*Verdun*	1	2	1
*L*. *interrogans*	*Icterohaemorrhagiae*	*Birkini*	*Birkin*	3	3	2
*L*. *interrogans*	*Australis*	*Fugis*	*Fudge*	3	3	2
*L*. *interrogans*	*Autumnalis*	*Autumnalis*	*Akiyami A*	3	3	2
*L*. *interrogans*	*Pyrogenes*	*Biggis*	*Biggis*	2	1	3
*L*. *interrogans*	*Canicola*	*Kuwait*	*136/2/2*	2	1	3
*L*. *interrogans*	*Canicola*	*Canicola*	*Hond Utrech*	2	1	3
*L*. *interrogans*	*Grippotyphosa*	*Grippotyphosa*	*Andaman*	2	1	3
*L*. *interrogans*	*Sejroe*	*Ricardi*	*Richardson*	2	1	3
NA	NA	NA	DNA of Leptospiraextracted from sera 1–13	1	2	1

The 42 *Leptospira* DNAs that were extracted from patient serum samples showed a Tm corresponding to that of the species *L*. *interrogans* using the primer pairs lfb1F/R and G1/G2.

Using VNTR-4 bis and VNTR-Lb5, the genotypes of 17 out of 42 (40.5%) DNA samples were identified. These 17 *Leptospira* DNA samples exhibited the same genotype, which was consistent with the strains L. *interrogans* sv. Copenhageni or Icterohaemorrhagiae.

Using VNTR-4 bis and VNTR-Lb5, the genotype determination was 100% when the Ct was ≤ 30 cycles (8250 copies/ml, data not shown) and 28.5% (10/35) when the Ct was >30. We then evaluated the integrity of three genotypable DNAs and three non-genotypable DNAs by performing a standard PCR targeting the *secY* gene. Agarose gel electrophoresis revealed multiple bands of different sizes for the non-genotypable DNAs resulting from nonspecific amplification, and a 202-bp band for the three genotypable DNAs. To understand how the DNA lost its integrity, we subjected three good quality DNAs to several freezing-defrosting processes. After each freezing-defrosting process, the migration of the amplified fragments showed progressive DNA degradation.

## Discussion

The main aim of this study was not to diagnose pathogenic leptospirosis infection but to describe a new genotyping method to identify *Leptospira* species and subspecies. For a better understanding of the epidemiology of leptospirosis in a region, information about the prevalent circulating serovars or genotypes is essential.

The first objective of the present study was to provide a rapid technique to discriminate between species of pathogenic *Leptospira* (only the four most frequent pathogenic leptospires were considered [45]), which is a necessary step for further levels of genotyping, i.e., at the serovar level. Using two sets of primers (G1/G2 and lfb1 R/F), the melting temperature profiles of the strains are able to accurately discriminate *L*. *interrogans*, *L*. *kirschneri*, *L*: *borgpetersenii*, *L*. *noguchii*, and the newly described *L*. *mayottensis* [33] with a specificity and reproducibility of 100% with less than 0.5% Tm coefficients of variation. Our method enabled us to accurately determine the infectious *Leptospira* species from DNA extracted from the blood of sick patients at Reunion Island. Previous methods for the identification of *Leptospira* species, such as PCR amplification followed by electrophoresis on a non-denaturing polyacrylamide gel [46] or the sequencing of partial 16SrDNAs [47], are time-consuming. In contrast, this method allows for species discrimination in a short time period. Recently described PCR methods for the diagnosis of acute infection [48] may have a lower end-point detection than our PCRs, consequently, a PCR-positive sample that has been identified with these highly sensitive methods might be unsuitable for characterization by melting temperature analysis. In a previous study, Merien et al. [35] used a similar melting curve analysis with the primer pairs lfb1 F/R, which facilitated the differentiation of the pathogenic species *L*. *interrogans*, *L*. *borgpetersenii*, and *L*. *kirschneri*, but not *L*. *noguchii* and *L*. *kirschneri*. In our study, the use of a second set of primers provided improved specificity. In a recent study, Feirrera et al [49] developed sets of species-specific probes with primers designed from ompL1 and secY with 100% analytical specificity for the species *L*. *interrogans*, *L*. *kirschneri*, *L*. *borgpetersenii*, and *L*. *noguchii*. These probes were specifically designed for these four species and do not allow for the detection and identification of the new species *L*. *mayottensis*.

Tulsiani et al. [31] applied a high-resolution melting curve analysis of random-amplified-polymorphic DNA to reference collection strains. MLVA has been previously proposed for typing the *L*. *interrogans*, *L*. *borgpetersenii*, and *L*. *kirschneri* strains [40,13]. In our study, the HRM analysis of the VNTR sequences was performed using ScreenClust HRM software. To the best of our knowledge, our study signifies the first time that *Leptospira* strains have been genotyped using VNTR primers. Using our method, we obtained a high Hunter-Gaston's discriminatory index (0.98) compared to a serological classification of all the reference strains tested. This high index (> 0.95) shows the genetic polymorphism within each tested species and validates the high sensitivity and specificity of auto-calling genotypes from HRM data using ScreenClust software.

The discriminatory index was relatively lower for *L*. *kirschneri* (0.87), which is potentially due to the decreased polymorphism of this species. Using MLVA, Salaün et al.[40] showed that the genetic diversity of *L*. *kirschneri* serovars was lower than *L*. *interrogans* serovars.

Using the ‘unsupervised analysis’ mode, HRM analysis using ScreenClust HRM software permitted the determination of clusters without genotypic knowledge. HRM analysis using ScreenClust HRM software also enabled the automated genotyping of unknown samples.

Although the performance measurements of our typing method [41] were excellent (reproducibility 100%, typeability 100%, discriminatory index 0.98), not all the strains (37/48, 77.1%) could be discriminated at the serovar level. The identification of *Leptospira* serovars by serological methods relies on surface-exposed lipopolysaccharides (LPS) and there is a poor correlation between serological and molecular typing methods that are not LPS-based. Like MLST, PFGE, and MLVA, our HRM analysis, which is based on DNA sequence analysis, did not always correlate with the serological characterization. The MLST, PFGE, and MLVA, and HRM methods have the ability to discriminate *Leptospira* strains at the subspecies level. However, depending on the technique used, distinct levels of discrimination can be obtained. In general, PFGE discrimination is superior to that of MLST, MLVA, and HRM analysis. For example, serovars Pomona and Guaratuba, which do not belong to the same serogroup, share the same ST [40,50] and HRM profiles but are distinguishable by PFGE [51]. For *L*. *kirschneri*, the genotype determined by HRM for serovars Bogvere and Djatzi was identical, but they had different profiles using MLVA and PFGE. MLST, PFGE, MLVA, and HRM cannot distinguish between strains belonging to the serovars Copenhageni and Icterohaemorrhagiae. Currently, no single molecular typing method is ideal and fulfils all the characteristics required to unambiguously determine if the isolates from numerous patients and/or animals are identical. However, HRM curve analysis is a simple and effective technique that does not require culture isolation and can be performed in a single test tube in less than two hours.

Our molecular investigation of the *Leptospira* DNA extracted from blood samples revealed that the *Leptospira* species responsible for 100% of the clinical cases in humans on Reunion Island was *L*. *interrogans*. In contrast, on the sister island of Mayotte, only 8.5% of the isolated strains belong to *L*. *interrogans*, and no cases were attributed to the serogroup Icterohaemorrhagiae [52]. In our study, the Reunion Island patient infecting strains were all clustered by ScreenClust HRM software using the VNTR-4bis and -Lb5 primers into the *L*. *interrogans* strain serogroups Icterohaemorrhagiae serovar Copenhageni or Icterohaemorrhagiae. We can therefore conclude that only one genotype has been involved in human symptomatic leptospirosis on Reunion Island since 2008. This result reflects the persistence of a serovar over time in a geographical area. These results are also confirmed by our previous studies on human patients. Using a serological method, the strains we isolated in culture in 1998 and 2012 were identified as belonging to the *L*. *interrogans* serogroup Icterohaemorrhagiae. Between 1998 and 2009, the serogroup Icterohaemorrhagiae was reported to represent 59.3% of the human cases diagnosed on Reunion Island using MAT [53].

## Conclusions

Our genotyping method was able to identify a change in genotype that was responsible for the clinical cases on Reunion Island. The unsupervised mode of ScreenClust HRM should also enable one to discover new genotypes that are present in clinical samples. Our genotyping method is limited by the number of leptospires in blood samples, wherein approximately 8250 copies/ml are necessary to obtain 100% typeability.

In this study, the use of HRM confirmed the predominance of a clonal subpopulation of *L*. *interrogans* as the cause of human leptospirosis on Reunion Island, suggesting that this method would be useful for epidemiological studies.
